# Supplementary material for: Traveling pulse emerges from coupled intermittent walks: A case study in sheep
Source: PLoS One. 2018 Dec 5;13(12):e0206817. doi: 10.1371/journal.pone.0206817 (PMC6281248; doi:10.1371/journal.pone.0206817)
Supplement: S1 Appendix — (PDF) [file pone.0206817.s005.pdf]

Traveling pulse emerges from coupled intermittent walks: a case study in sheep

Manon Azaïs[1], Stéphane Blanco[2], Richard Bon[1], Richard Fournier[2], Marie-Hélène Pillot[1], Jacques Gautrais[1*]

1 Centre de Recherches sur la Cognition Animale (CRCA), Centre de Biologie Intégrative (CBI), Université de Toulouse; CNRS, UPS, France.
2 LaPlaCE, Université de Toulouse; CNRS, UPS, France.

* jacques.gautrais@univ-tlse3.fr

S1 Appendix : Analytical solution in the steady regime for the minimal model

Macroscopic model

Let $A(x,t)$ and $I(x,t)$ denote respectively the density of active (moving) and inactive (stopped) sheep at location x at time t. They evolve according to:

$$\begin{cases} \partial_t I(x,t) &= -K_A(x,t)I(x,t) + K_I(x,t)A(x,t) \\ \partial_t A(x,t) + v\partial_x A(x,t) &= +K_A(x,t)I(x,t) - K_I(x,t)A(x,t) \end{cases} \tag{1}$$

where $K_A(x,t)$ and $K_I(x,t)$ are respectively the conversion rates from stopped-to-moving (activation) and moving-to-stopped (inactivation) at location x at time t, which depend on A and I according to:

$$\begin{cases} K_A(x,t) = \mu_{_A} + \alpha_{_A} \left[\int_x^{\infty} A(u,t)du\right]^{\beta_A} \left[N - \int_x^{\infty} A(u,t)du\right]^{-\gamma_A} \\ K_I(x,t) = \mu_{_I} + \alpha_{_I} \left[\int_{-\infty}^{x} I(u,t)du\right]^{\beta_I} \left[N - \int_{-\infty}^{x} I(u,t)du\right]^{-\gamma_I} \end{cases} \tag{2}$$

with $N = \int_{-\infty}^{\infty} A(u,t) + I(u,t)du$ is the total amount of sheep (which is conserved in time), and parameters are those given in the individual-based model. This description in density is the direct translation of the IBM expressions in the limit of continuum theory.

Let now consider the classical macroscopic descriptors $\eta(x,t)$, the sum density of sheep (moving and stopped) at location x at time t, and the corresponding flow $j(x,t)$, defined by:

$$\begin{cases} \eta(x,t) = A(x,t) + I(x,t) \\ j(x,t) = v\ A(x,t) + 0\ I(x,t) \end{cases} \tag{3}$$

Introducing $\beta(x,t)$, the moving fraction at location x at time t, defined by:

$$\beta(x,t) = \frac{A(x,t)}{A(x,t) + I(x,t)} = \frac{A(x,t)}{\eta(x,t)} \tag{4}$$

we have $A(x,t) = \beta(x,t)\eta(x,t)$, so that $j(x,t) = v\ \beta(x,t)\eta(x,t)$.

The macroscopic description in Eq (3) can then be equivalently expressed by:

$$\begin{cases} \eta(x,t) = A(x,t) + I(x,t) \\ \beta(x,t)\eta(x,t) = A(x,t) \end{cases} \tag{5}$$

which we will use from now on. The evolution of these macroscopic descriptors is derived from Eq (1), summing the two evolutions to obtain η, and using only the second as for $\beta\eta$, leading to:

$$\begin{cases} \partial_t \eta + v\ \partial_x(\beta\eta) &= 0 \\ \partial_t(\beta\eta) + v\ \partial_x(\beta\eta) &= K_A(1-\beta)\eta - K_I\beta\eta \end{cases} \tag{6}$$

in which dependencies to (x,t) have been omitted for the sake of clarity, and where K_A and K_I are now expressed in macroscopic terms, following:

$$\begin{cases} K_A(x,t) = \mu_A + \alpha_A\ \left[A^+(x,t)\right]^{\beta_A} \quad \left[N - A^+(x,t)\right]^{-\gamma_A} \\ K_I(x,t) = \mu_I + \alpha_I\ \left[I^-(x,t)\right]^{\beta_I} \quad \left[N - I^-(x,t)\right]^{-\gamma_I} \end{cases} \tag{7}$$

with $A^+(x,t)$ the quantity of moving sheep ahead of x, and I^- the quantity of stopped sheep behind x :

$$\begin{cases} A^+(x,t) = \displaystyle\int_x^{\infty} \beta(u,t)\eta(u,t)du \\ I^-(x,t) = \displaystyle\int_{-\infty}^{x} (1-\beta(u,t))\eta(u,t)du \end{cases} \tag{8}$$

Macroscopic minimal model

We are looking for steady regimes for the minimal model where $\gamma_\bullet = 0$ and $\beta_\bullet = 1$.

The macroscopic minimal model reads:

$$
\begin{cases}
\partial_t \eta(x,t) + v\ \partial_x(\beta(x,t)\eta(x,t)) = 0 \\
\partial_t(\beta(x,t)\eta(x,t)) + v\ \partial_x(\beta(x,t)\eta(x,t)) = \eta(x,t) \times \\
\qquad \Big[(1-\beta(x,t))(\mu_A + \alpha_A \int_x^{\infty} \beta(u,t)\eta(u,t)du) \\
\qquad - \beta(x,t)(\mu_I + \alpha_I \int_{-\infty}^{x} (1-\beta(u,t))\eta(u,t)du)\Big]
\end{cases}
\tag{9}
$$

summarized by:

$$
\begin{cases}
\partial_t \eta + v\ \partial_x(\beta\eta) = 0 \\
\partial_t(\beta\eta) + v\ \partial_x(\beta\eta) = \eta\Big[(1-\beta)K_A - \beta K_I\Big]
\end{cases}
\tag{10}
$$

where K_A and K_I are :

$$
\begin{cases}
K_A(x,t) = \mu_A + \alpha_A\ A^+(x,t) \\
K_I(x,t) = \mu_I + \alpha_I\ I^-(x,t)
\end{cases}
\tag{11}
$$

with $A^+(x,t)$ the quantity of moving sheep ahead of x, and I^- the quantity of stopped sheep behind x :

$$
\begin{cases}
A^+(x,t) = \int_x^{\infty} \beta(u,t)\eta(u,t)du \\
I^-(x,t) = \int_{-\infty}^{x} (1-\beta(u,t))\eta(u,t)du
\end{cases}
\tag{12}
$$

This expression gives the evolution of the density of sheep in a fixed frame (x,t), attached to the field (hereafter the field frame).

Non Homogeneous Steady Regime

Numerical simulations suggest the existence of a non homogeneous steady state with the form of a propagating wave. Here, we characterize this state.

Having in mind a solution in the form of a wave propagation, we will rewrite the system in another frame (y,t), which is moving at a constant speed c relatively to the field frame, with coordinates:

$$
\begin{cases}
y = R(x,t) = x - ct \\
t = Q(x,t) = t
\end{cases}
\tag{13}
$$

with coincidence of the two frames at initial time. We then have, for any $g(y,t) = f(R(x,t),Q(x,t))$:

$$
\begin{cases}
\partial_x f = \partial_x R\ \partial_y g + \partial_x Q\ \partial_t g \quad = \partial_y g \\
\partial_t f = \partial_t R\ \partial_y g + \partial_t Q\ \partial_t g \quad = \partial_t g - c\ \partial_y g
\end{cases}
\tag{14}
$$

Denoting $\tilde{\eta}(y,t) = \eta(R(x,t),Q(x,t))$ the density of sheep, and $\tilde{\beta}(y,t) = \beta(R(x,t),Q(x,t))$ the moving fraction in the moving frame, we then have from Eq 10:

$$\begin{cases} \partial_t\tilde{\eta} - c\ \partial_y\tilde{\eta} + v\ \partial_y(\tilde{\beta}\tilde{\eta}) = 0 \\ \partial_t(\tilde{\beta}\tilde{\eta}) - c\ \partial_y(\tilde{\beta}\tilde{\eta}) + v\ \partial_y(\tilde{\beta}\tilde{\eta}) = \tilde{\eta}\Big[(1-\tilde{\beta})\widetilde{K}_A - \tilde{\beta}\widetilde{K}_I\Big] \end{cases} \tag{15}$$

where $\widetilde{K}_A$ and $\widetilde{K}_I$ are:

$$\begin{cases} \widetilde{K}_A(y,t) = \mu_A + \alpha_A \displaystyle\int_y^{\infty} \tilde{\beta}(u,t)\tilde{\eta}(u,t)du \\ \widetilde{K}_I(y,t) = \mu_I + \alpha_I \displaystyle\int_{-\infty}^{y} (1-\tilde{\beta}(u,t))\tilde{\eta}(u,t)du \end{cases} \tag{16}$$

If steady states exist, they obey (in the moving frame):

$$\begin{cases} -c\ \partial_y\tilde{\eta} + v\ \partial_y(\tilde{\beta}\tilde{\eta}) = 0 \\ -c\ \partial_y(\tilde{\beta}\tilde{\eta}) + v\ \partial_y(\tilde{\beta}\tilde{\eta}) = \tilde{\eta}\Big[(1-\tilde{\beta})\widetilde{K}_A - \tilde{\beta}\widetilde{K}_I\Big] \end{cases} \tag{17}$$

that we rewrite as:

$$\begin{cases} -c\ n' + v\ (bn)' = 0 \\ -c\ (bn)' + v\ (bn)' = n\Big[(1-b)(\mu_A + \alpha_A A^+) - b(\mu_A + \alpha_A I^-)\Big] \end{cases} \tag{18}$$

where steady states (n,b) are such that $n(y) = \tilde{\eta}(y,t)$ and $b(y) = \tilde{\beta}(y,t)\ \forall t$, prime denotes regular derivative with respect to y, and

$$\begin{cases} A^+ = \displaystyle\int_y^{\infty} b(u)n(u)du \\ I^- = \displaystyle\int_{-\infty}^{y} (1-b(u))n(u)du \end{cases} \tag{19}$$

Propagation speed

A pure propagation of a wave would imply a pure advection of a non homogenous profile of n. In the static frame, this would imply that the first equation in Eq.6 is a pure advection, meaning:

$$\partial_t\eta + v\ \partial_x(\beta\eta) = \partial_t\eta + v\ \partial_x\eta = 0 \tag{20}$$

This can occur only when $\partial_x\beta = 0$, meaning an homogeneous profile for β, or equivalently, in the moving frame, $b(y) = b_s,\ \forall y$.

Plugging into the first equation of Eq.18, this translates into:

$$- c\ n' + v\ (b_s n)' = -c\ n' + v\ b_s\ n' = 0 \tag{21}$$

yielding

$$c = b_s v \tag{22}$$

meaning, consistently, that the density profile would propagate at a speed $b_s v$ equal to the average speed (the moving fraction times the individual speed).

Wave profile for the density $n(y)$

What would be the steady state density profile in this particular moving frame? Second equation in Eq (18) becomes:

$$\begin{aligned} vb_s(1-b_s)n' = n\Big[&(1-b_s)\left(\mu_A + \alpha_A b_s \int_y^\infty n(u)du\right) \\ &- b_s\left(\mu_I + \alpha_I(1-b_s)\int_{-\infty}^y n(u)du\right)\Big] \end{aligned} \tag{23}$$

Rearranging terms, we get:

$$\begin{aligned} vb_s(1-b_s)n' =& n\left[(1-b_s)\mu_A - b_s\mu_I\right] \\ &+ nb_s(1-b_s)\left[\alpha_A N^+ - \alpha_I N^-\right] \end{aligned} \tag{24}$$

with $N^+ = \int_y^\infty n(u)du$ and $N^- = \int_{-\infty}^y n(u)du$.

Deriving with respect to y, we get:

$$\begin{aligned} vb_s(1-b_s)n'' =&\ n'\left[(1-b_s)\mu_A - b_s\mu_I\right] \\ +&\ n'b_s(1-b_s)\left[\alpha_A N^+ - \alpha_I N^-\right] \\ +&\ nb_s(1-b_s)\left[\alpha_A(-n) - \alpha_I(n)\right] \\ =&\ n'\left[(1-b_s)\mu_A - b_s\mu_I\right] \\ +&\ n'b_s(1-b_s)\left[\alpha_A N^+ - \alpha_I N^-\right] \\ -&\ n^2 b_s(1-b_s)\left[\alpha_A + \alpha_I\right] \end{aligned} \tag{25}$$

Multiplying by n, we obtain:

$$\begin{aligned} vb_s(1-b_s)n''n =&\ n'n\left[(1-b_s)\mu_A - b_s\mu_I\right] \\ +&\ n'\left\{nb_s(1-b_s)\left[\alpha_A N^+ - \alpha_I N^-\right]\right\} \\ -&\ n^3 b_s(1-b_s)\left[\alpha_A + \alpha_I\right] \end{aligned} \tag{26}$$

From Eq.24, we extract the term in {} in Eq.26 :

$$nb_s(1-b_s)\left[\alpha_A N^+ - \alpha_I N^-\right] = vb_s(1-b_s)n' - n\left((1-b_s)\mu_A - b_s\mu_I\right) \tag{27}$$

that we plug back into Eq.26, and we obtain:

$$\begin{aligned}
vb_s(1-b_s)n''n = & \quad n'n\left[(1-b_s)\mu_A - b_s\mu_I\right] \\
+ & \quad n'\left[vb_s(1-b_s)n' - n\left((1-b_s)\mu_A - b_s\mu_I\right)\right] \\
- & \quad n^3 b_s(1-b_s)\left[\alpha_A + \alpha_I\right] \\
= & \quad n'n\left[(1-b_s)\mu_A - b_s\mu_I\right] \\
+ & \quad (n')^2 vb_s(1-b_s) \\
- & \quad n'n\left[(1-b_s)\mu_A - b_s\mu_I\right] \\
- & \quad n^3 b_s(1-b_s)\left[\alpha_A + \alpha_I\right] \\
= & \quad (n')^2 vb_s(1-b_s) \\
- & \quad n^3 b_s(1-b_s)\left[\alpha_A + \alpha_I\right]
\end{aligned} \tag{28}$$

finally yielding:

$$n'^2 - nn'' - \frac{\alpha_A + \alpha_I}{v} n^3 = 0 \tag{29}$$

Let $\xi = (\alpha_A + \alpha_I)/v$.
A solution to Eq.29 compatible with a wave peak at $y = 0$ (i.e. $n'(0) = 0$) is:

$$n(y) = \frac{K}{2\xi}\text{sech}^2\left((1/2)\sqrt{K}y\right) \tag{30}$$

with K a constant. To solve for this constant, we consider the constraint that $\int_{-\infty}^{+\infty} n(u)du = \text{N}$, the number of sheep, is conserved.
Since

$$\int_{-\infty}^{+\infty} \text{sech}^2\left(ky\right) dy = 2/k \tag{31}$$

we then have:

$$\int_{-\infty}^{+\infty} n(u)du = \int_{-\infty}^{+\infty} du \frac{K}{2\xi}\text{sech}^2\left((1/2)\sqrt{K}u\right) = \frac{2\sqrt{K}}{\xi} = \text{N} \tag{32}$$

so that

$$K = (1/4)\ \text{N}^2\ \xi^2 \tag{33}$$

Finally, the profile for the density, in the frame moving at speed $\beta_s v$, is then:

$$n(y) = \frac{1}{2}\mathrm{N}\gamma\mathrm{sech}^2\left(\gamma y\right) \tag{34}$$

with

$$\gamma = \frac{\mathrm{N}(\alpha_A + \alpha_I)}{4v} \tag{35}$$

We note that this solution $n(y)$ for this steady state *profile* is independent from the value b_s as well as from the spontaneous activation/inactivation parameters μ_A and μ_B. We see below that those parameters are only involved in the propagation speed of this profile.

Value of the moving fraction b_s

The solution above is compatible with the model only if the corresponding moving fraction b_s is such that $b_s \in [0..1]$.

This solution with a wave peak at $y = 0$ is compatible with only one value for b_s, since we must have that Eq.23 holds at $y = 0$, namely :

$$\begin{aligned} v b_s(1-b_s)n'(0) = n(0)\Big[&(1-b_s)\left(\mu_A + \alpha_A b_s \int_0^\infty n(u)du\right) \\ &- b_s\left(\mu_I + \alpha_I(1-b_s)\int_{-\infty}^0 n(u)du\right)\Big] \end{aligned} \tag{36}$$

With $n'(0) = 0$ and $n(0) > 0$, b_s must then be solution to:

$$(1-b_s)\left(\mu_A + \alpha_A b_s \int_0^\infty n(u)du\right) - b_s\left(\mu_I + \alpha_I(1-b_s)\int_{-\infty}^0 n(u)du\right) = 0 \tag{37}$$

The wave solution being symetrical around $y = 0$, we have:

$$\int_0^\infty n(u)du = \int_{-\infty}^0 n(u)du = N/2 \tag{38}$$

so that b_s must be solution to:

$$(1-b_s)\left(\mu_A + \alpha_A b_s N/2\right) - b_s\left(\mu_I + \alpha_I(1-b_s)N/2\right) = 0 \tag{39}$$

i.e.

$$b_s^2\left[N/2(-\alpha_A + \alpha_I)\right] + b_s\left[N/2(\alpha_A - \alpha_I) - (\mu_A + \mu_I)\right] + \mu_A = 0 \tag{40}$$

i.e.

$$C\, b_s^2 - \left(C - (\mu_A + \mu_I)\right)\, b_s - \mu_A = 0 \tag{41}$$

with $C = \frac{N}{2}(\alpha_A - \alpha_I)$.

We note that, in the symetrical case where $\alpha_A = \alpha_I$, we have $b_s = \mu_A/(\mu_A + \mu_I)$.

In other cases, we check that :

$$(C - \mu_A - \mu_I)^2 + 4\mu_A C = (C + \mu_A - \mu_I)^2 + 4\mu_A \mu_I \tag{42}$$

which is always positive because parameter rates are positive or null ; hence solutions to Eq.40 are real.

The correct solution is actually always given by the root:

$$b_s = \frac{(C - \mu_A - \mu_I) + \sqrt{(C - \mu_A - \mu_I)^2 + 4\mu_A C}}{2C} \tag{43}$$

Of course, C can be any real value (negative or positive), depending on $\alpha_A - \alpha_I$. However, we have:

$$\begin{aligned} \lim_{C \to -\infty} b_s(C) &= 0 \\ \lim_{C \to +\infty} b_s(C) &= 1 \\ b_s'(C) &> 0 \quad \forall C \end{aligned} \tag{44}$$

so that there always is a solution $b_s \in [0..1]$ for any set of parameters (see Fig. S3 for some illustration).

Complete solution in the field frame

A non homogeneous steady regime is then a propagating wave with a spatial profile in sech^2 with:

$$\begin{cases} \eta^s(x,t) = \frac{\text{N}}{2}\frac{\text{N}(\alpha_A + \alpha_I)}{4v}\text{sech}^2\left(\frac{\text{N}(\alpha_A + \alpha_I)}{4v}\ (x - b_s^* vt)\right) \\ \beta^s(x,t) = b_s^* \\ \qquad = \frac{\left(\frac{N}{2}(\alpha_A - \alpha_I) - \mu_A - \mu_I\right) + \sqrt{\left(\frac{N}{2}(\alpha_A - \alpha_I) - \mu_A - \mu_I\right)^2 + 4\mu_A \frac{N}{2}(\alpha_A - \alpha_I)}}{N(\alpha_A - \alpha_I)} \end{cases} \tag{45}$$

when $\alpha_A \neq \alpha_I$.

In the symetrical case where $\alpha_A = \alpha_I$, the steady state solution simplifies to:

$$\begin{cases} \eta^s(x,t) = \frac{N}{2}\frac{N\alpha}{2v}\ \text{sech}^2(\frac{N\alpha}{2v}\ (x - b_s^* vt)) \\ \beta^s(x,t) = b_s^* = \mu_A/(\mu_A + \mu_I) \end{cases} \tag{46}$$
